# Health-related quality of life among Chinese adolescent girls with Dysmenorrhoea

**DOI:** 10.1186/s12978-018-0540-5

**Published:** 2018-05-16

**Authors:** Cho Lee Wong

**Affiliations:** The Nethersole School of Nursing, Faculty of Medicine, The Chinese University of Hong Kong, Room 824, Esther Lee Building, Shatin, New Territories Hong Kong

**Keywords:** Adolescent, Dysmenorrhoea, Quality of life

## Abstract

**Background:**

Primary dysmenorrhoea is common in girls who have begun menstruating. However, few studies have examined its effect on the quality of life of a young population. The study aimed to evaluate the quality of life of adolescent girls with dysmenorrhoea in Hong Kong.

**Methods:**

The study adopted a cross-sectional descriptive approach. A convenience sample of 653 girls aged 13 to 19 years old was recruited from three secondary schools in Hong Kong. The 36-item Short-Form Health Survey was used to examine the health-related quality of life of the participants. The severity of dysmenorrhoea was assessed using a 10-point visual analog scale.

**Results:**

Girls suffering from dysmenorrhoea reported high pain prevalence and intensity. However, the majority of girls with dysmenorrhoea did not seek medical advice (93.2%) or self-medicate (82%). The role-physical, bodily pain, general health and social functioning domain scores of girls with dysmenorrhoea were significantly lower than those without dysmenorrhoea. Moreover, girls with severe dysmenorrhoea had a significantly lower quality of life in the bodily pain domain than those with mild and moderate forms of condition.

**Conclusions:**

Findings suggest that dysmenorrhoea is highly prevalent among adolescent girls in Hong Kong. Girls may suffer severe pain, which degrades their quality of life.

## Plain English summary

Dysmenorrhoea not only affects girls’ physical health, but also affects their daily lives and social activities. These effects, in turn, likely affect the emotional and social functions of girls with dysmenorrhoea. Some studies have examined quality of life as a specific construct among university students and women with dysmenorrhoea. However, the influence of dysmenorrhoea on the quality of life of adolescent Chinese girls has been poorly studied. This study describes and compares the quality of life of adolescent Chinese girls with or without dysmenorrhoea. A total of 653 girls were recruited from three secondary schools in Hong Kong. The prevalence of dysmenorrhoea was 65.5% in this group. Over 90% of the participants did not seek medical advice, and some participants reported self-medicating with an incorrect dose or unknown medication. In addition, girls with dysmenorrhoea have significantly lower quality of life than those without dysmenorrhoea. Healthcare professionals should be made aware that adolescent girls who suffer from dysmenorrhoea are likely to have a lower quality of life. Adolescent girls should be screened for dysmenorrhoea symptoms and promptly provided with appropriate interventions. Moreover, the understanding of self-medication among Chinese adolescent girls should be improved. Appropriate educational interventions related to dysmenorrhoea, with the correct administration of self-medication as an essential component, should be developed and provided.

## Background

Primary dysmenorrhoea is one of the most common menstrual problems among adolescent girls [1] and is defined as painful menstruation in the absence of abnormal pelvic anatomy and ovulation [2]. It often commences six to twelve months post-menarche, and its symptoms appear a few hours prior to or with the onset of menstrual flow. Symptoms of dysmenorrhoea include cramping pain that often radiates to the lower back or anterior thigh from the lower abdomen [3]. Pain is most intense during the first 24 to 36 h and lasts for two to three days [4]. Some girls may experience nausea, vomiting, headache, loose bowel movements or dizziness as well [5]. Dysmenorrhoea is highly prevalent among adolescent girls: worldwide, approximately 20 to 90% of adolescent girls suffer from dysmenorrhoea [6–8]. Its prevalence progressively decreases with age [9]. To date, no extensive epidemiological study has investigated the prevalence of dysmenorrhoea among adolescent girls in Hong Kong. One local study indicated that 68.7% of 5609 secondary-school girls aged 11 to 20 suffer from dysmenorrhoea [10].

Dysmenorrhoea affects adolescent girls’ academic, social and physical activities [3, 11]. Other studies have quantified the effect of dysmenorrhoea in terms of days of lost work or school absenteeism [8, 12–15]. Dysmenorrhoea results in the loss of 600 million working hours and two billion dollars’ worth of productivity leach year in the United States [12]. Dysmenorrhoea also negatively affects sufferers’ quality of life [16–18]. A study in Hong Kong utilised the Short-Form Health Survey (SF-36) to compare the effects of different menstrual problems, such as dysmenorrhoea, amenorrhea (absence of a menstrual period in a girl with reproductive age), menorrhagia (periods with abnormally heavy or prolonged bleeding) and eumenorrhoeic (normal periods), on 235 adolescents [18]. Results showed that adolescents with dysmenorrhoea had the lowest quality of life scores in the bodily pain domain. Moreover, their scores in the general health and social functioning domains were significantly lower than those of girls without menstrual problems. However, the above results should be treated with caution given the small sample size of subjects with dysmenorrhoea (*n* = 43).

Currently, few studies have examined quality of life as a specific construct among university students and women with dysmenorrhoea [16, 19]. However, the influence of dysmenorrhoea on the quality of life among adolescent Chinese girls has been poorly studied. Therefore, this study aimed to examine the effects of dysmenorrhoea on the quality of life of adolescent girls in Hong Kong. In this study, dysmenorrhoea was defined in accordance with a review conducted by the World Health Organization [1]. It was defined as painful menses within the last three months [8]. It is hypothesised that adolescent girls with dysmenorrhoea will report a lower quality of life than those without dysmenorrhoea.

## Methods

### Sample and setting

The study sample was recruited from Hong Kong secondary schools and was randomly selected. The sample was recruited from secondary schools given that all girls in their early adolescence are in Forms 1 to 3 in accordance with the compulsory education policy of Hong Kong; moreover, according to a previous government report, approximately 85% of students aged 15–16 are in Forms 4 or 5 [20]. Boys-only and international schools were excluded.

Given that the mean age of menarche among Hong Kong adolescent girls is 12.3 [10], and girls experience dysmenorrhoea at six to twelve months after menarche when ovulatory cycles are established [21], a convenience sample of students from Forms 2 to 7 (13 to 19 years old) were recruited to avoid having large numbers of pre-menarche girls. Information about dysmenorrhoea was collected from those who indicated that they had experienced dysmenorrhoea within the last three month [8, 22]. Girls who (1) were pre-menarche, (2) had a previous history of gynecological disease or surgery or (3) had a severe condition such as heart or malignant disease, were excluded.

### Outcome measures

#### The demographic and menstrual characteristics questionnaire

A 19-item questionnaire for the demographic and menstrual characteristics of adolescents was developed by the researcher given the lack of an appropriate questionnaire. The questionnaire was developed on the basis of available literature [23].

The Pain Visual Analogue Scale (PVAS) was used to assess the average intensity of dysmenorrhoea pain experienced within the last three months [24, 25]. The PVAS consists of a 10 cm horizontal line with verbal descriptors at either end (0 = “no pain”; 10 =“worst possible pain”). Subjects were instructed to place a cross on the line that represent the average intensity of their pain. Scores ranged from 0 to 10, with high scores indicating high pain intensity. The scale has well-established reliability and validity with a Cronbach’s alpha of 0.94 [26]. Scores between 1 and 3.9 were classified as mild dysmenorrhoea, those between 4 and 7.9 as moderate dysmenorrhoea, and those between 8 and 10 as severe dysmenorrhoea [27].

#### Health-related quality of life

The HRQOL of the participants were assessed using the 36-item SF36 [28]. The form consists of 36 questions that measure eight HRQOL domains: physical functioning, role-physical, bodily pain, general health, vitality, social functioning, role-emotional and mental health. Item scores in each domain were summed to yield domain scores, which were transformed into a range of 0 to 100. A high score indicated superior HRQOL. The eight domain scores were summed to form the physical and mental component summary scores. The Chinese (HK) SF-36v2 used in this study had undergone validity testing and was normalised to the general Chinese population in Hong Kong [29, 30].

### Procedure

A letter was sent to schools inviting their students to join the study. Students who showed interest received written information. In a classroom or hall setting, a female research assistant explained details of the study. Girls who agreed to participate were asked to complete the informed consent forms and questionnaires, which included the structured demographic and menstrual characteristics and the Chinese (HK) version SF-36v2 questionnaires. During the data collection process, the researcher answered queries about the questionnaires.

### Statistical analyses

SPSS 22.0 (SPSS, Chicago, IL) was used to analyse data and descriptive statistics to present the participants’ characteristics and HRQQL scores. For each outcome variables, an independent sample t-test or chi-square test was performed to identify differences between girls with or without dysmenorrhoea. Linear regressions were used to compare HRQOL between the two groups with adjustments for significantly different socio-demographics. A one-way analysis of variance or a Kruskal-Wallis test was used to compare menstrual characteristics and quality of life scores among the mild, moderate and severe dysmenorrhoea groupings. All statistical tests were two-tailed, and the level of statistical significance was set at 5%.

### Ethical approval

Ethical approval from the study institution and permission to use the study instruments were obtained. This study was carried out in accordance with the Declaration of Helsinki. Participants were informed that their participation was voluntary, and they could withdraw from the study at any time. They were also told that all the information collected would be kept confidential.

## Results

Six secondary schools were approached and three agreed to participate in the study with a participation rate of 50%. A total of 704 girls from the three schools participated, 51 of them were excluded (two with gynecological disease, 21 were pre-menarche and 28 produced incomplete item responses). The final sample consisted of 653 girls.

### Demographic and menstrual characteristics

The ages of the participants ranged from 13 to 19 years (mean = 15.67, SD = 1.54). The average body mass index of the participants was 19.00 (SD = 2.63). Nearly 40% (*n* = 251) had a family income of more than HK$20,000. Their age at menarche ranged from 10 to 16 (mean = 12.07, SD = 2.23). Over 70% (*n* = 504) of the participants had a menstrual cycle of between 21 and 35 days, with a mean menstruation period of 5.60 days (SD = 1.08). A majority had moderate menstruation (*n* = 528). The prevalence of dysmenorrhoea in the recruited group was 65.5% (*n* = 428). Participants with dysmenorrhoea were significantly older than those without dysmenorrhoea (*p* < 0.001). The demographics and menstrual characteristics of the participants are shown in Table 1.

**Table 1:** Demographic and menstrual characteristics of the participants

	Total sample (*n* = 653)	With dysmenorrhea (n = 428)	Without dysmenorrhea (*n* = 225)	p-value
Age (years)				
Mean (SD)	15.67 (1.54)	15.82 (1.56)	15.40 (1.50)	< 0.001^a^
Height (cm)				
Mean (SD)	159.32 (5.79)	159.39 (5.66)	159.16 (6.04)	0.62^a^
Weight (kg)				
Mean (SD)	48.25 (7.24)	48.10 (7.14)	48.53 (7.44)	0.48^a^
BMI				
Mean (SD)	19.00 (2.63)	18.91 (2.47)	19.12 (2.91)	0.25^a^
Family income (HK$)				
< 5000	20 (3.1)	12 (2.8)	8 (3.6)	0.27^b^
5001–10,000	136 (20.8)	80 (18.7)	56 (24.9)	
10,001–20,000	246 (37.7)	166 (38.8)	80 (35.6)	
20,001–30,000	146 (22.4)	94 (22.0)	52 (23.1)	
> 30,001	105 (16.1)	76 (17.8)	29 (12.9)	
Age at menarche (years)				
Mean (SD)	12.07 (2.23)	12.06 (1.00)	12.14 (1.07)	0.33^a^
Menstrual cycle (days)				
16–20	47 (7.2)	29 (6.8)	18 (8.0)	0.44^b^
21–25	137 (21.0)	97 (22.7)	40 (17.8)	
26–30	262 (40.1)	165 (38.6)	97 (43.1)	
31–35	105 (16.1)	74 (17.3)	31 (13.8)	
36-40	45 (6.9)	31 (7.2)	14 (6.2)	
≥ 40	57 (8.8)	32 (7.5)	25 (11.1)	
Menstruation duration (days)				
Mean (SD)	5.60 (1.08)	5.60 (1.10)	5.61 (1.12)	0.93^a^
Amount of menstruation				
Small	42 (6.4)	24 (5.6)	18 (8.0)	0.30^b^
Moderate	528 (80.9)	345 (80.6)	183 (81.3)	
Large	83 (12.7)	59 (13.8)	24 (10.7)	

Data is presented as frequencies (percentages)

*SD* standard deviation

^a^ independent sample t-test

^b^ chi-square test

### Menstrual characteristics of girls with dysmenorrhoea

Participants with dysmenorrhoea reported an average pain intensity over the previous three menstrual cycles of 5.43 (SD = 2.13). Of the participants, 27.1% reported mild pain, 60.8% reported moderate pain and 12.1% reported severe pain. The most common area of pain was the lower abdomen (83.9%) and lumbar region (6.1%). Fatigue (37.5%) and breast tenderness (16.1%) were other common complaints. Nearly 40% of the subjects first experienced dysmenorrhoea six months to a year after menarche. More than half (51.6%) of the participants reported that the onset of dysmenorrhoea occurred on the first day of their menstrual cycle, and 34.8% reported that their dysmenorrhoea symptoms disappeared on the third day of their cycle. A total of 239 girls (55.8%) reported that dysmenorrhoea only slightly affected their daily activities. Approximately 16% (*n* = 68) of the participants were absent from class in the current half year because of dysmenorrhoea. Approximately half of the participants (56.8%) had received menstrual education. The person most commonly consulted on the subject of dysmenorrhoea was their mother (*n* = 201) or a friend (*n* = 73). The menstrual characteristics of adolescent girls with dysmenorrhoea are shown in Table 2.

**Table 2:** Menstrual characteristics of adolescent girls with dysmenorrhea

	With dysmenorrhea (*n* = 428)
Pain intensity	
Mean (SD)	5.43 (2.13)
Area of dysmenorrheal pain	
Upper abdomen	17 (4.0%)
Lower abdomen	359 (83.9%)
Pelvic region	17 (4.0%)
Lumbar region	26 (6.1%)
Inner thigh	5 (1.2%)
Other	4 (0.9%)
Other discomforts	
Breast tenderness	85 (16.1%)
Swelling of lower limbs	17 (3.2%)
Fatigue	197 (37.5%)
Nausea	35 (6.7%)
Headache	43 (8.2%)
Diarrhea	33 (6.3%)
Anxiety	38 (7.2%)
Palpitations	4 (0.8%)
Skin problems	57 (10.8%)
Other	17 (3.2%)
First experience of dysmenorrhea	
In the first menarche	82 (19.2%)
6 months to a year after menarche	161 (37.6%)
1 to 2 years after menarche	93 (21.7%)
2 years after menarche	68 (15.9%)
Other/ forgotten	24 (5.6%)
Onset of dysmenorrhea	
Day before menstrual cycle	109 (25.5%)
First day of cycle	221 (51.6%)
Second day of cycle	80 (18.7%)
Other	18 (4.2%)
End of dysmenorrhea	
First day of menstrual cycle	54 (12.6%)
Second day of menstrual cycle	135 (31.5%)
Third day of menstrual cycle	149 (34.8%)
Fourth day of menstrual cycle	56 (13.1%)
Fifth day of menstrual cycle	22 (5.1%)
Others	12 (2.8%)
Limitation of daily activities due to dysmenorrhea	
None	52 (12.1%)
Slightly affected	239 (55.8%)
Moderately affected	108 (25.2%)
Seriously affected	29 (6.8%)
Absence from class in this half year due to dysmenorrhea (days)	
0	360 (84.1%)
1	43 (10%)
2	15 (3.5%)
3	6 (1.4%)
≥ 4	4 (0.9%)
Received menstrual education	
No	185 (43.2%)
Yes	243 (56.8%)
Others consulted about dysmenorrheal	
No one	174
Mother	201
Father	3
Sister	42
Friend	73
Teacher	4
Nurse	2
Medical consultation for dysmenorrhea	
None	399 (93.2%)
Chinese practitioner	9 (2.1%)
Modern practitioner	20 (4.7%)
Self-medication for dysmenorrhea in the last 3 months	
None	351 (82%)
Chinese herbs/ medicine	6 (1.4%)
Modern medicine	71 (16.6%)

A majority of girls did not seek medical advice (93.2%) or self-medicate (82%) during the last three cycles, whereas 16.6% took modern medicine and 1.4% took Chinese herbs or traditional remedies for their pain (Table 2). The medication and dosage the participants used were revealed through open-ended questions. A total of 62 participants took one to three tablets of paracetamol, and one took poston, a non-steroidal anti-inflammatory drug. One girl reported taking a few tablets of paracetamol, which is used to treat pain and fever, each time she experienced pain. One girl mentioned using famotidine, which is used to inhibit stomach acid production. Other girls reported taking one tablet of an unknown medication. Among those using Chinese herbs or remedies, two took Bak Foong pill, which is used to relieve menstrual discomfort. Three took Gu Sao pill to relieve menstrual discomfort. However, one girl was unable to name the medication she took.

### Menstrual characteristics of adolescent girls with different severities of dysmenorrhoea

Compared with adolescent girls with mild or moderate dysmenorrhoea, girls with severe dysmenorrhoea were significantly more likely to have higher pain intensity (*p* < 0.001), more limited daily activities (*p* = 0.002), and a higher absence rate (p < 0.001). They were more likely to consult others (p < 0.001) and self-medicate (*p* < 0.001). However, they were less likely to have received menstrual education (*p* = 0.016) (Table 3).

**Table 3:** Menstrual characteristics of adolescent girls with different severity rates of dysmenorrhea

	Mild dysmenorrhea (*n* = 116)	Moderate dysmenorrhea (*n* = 260)	Severe dysmenorrhea (n = 52)	p-value
Pain intensity				
Mean (SD)	2.75 (0.78)	5.94 (1.10)	8.87 (0.81)	< 0.001^a^
Limitation of daily activities due to dysmenorrhea				
None	24 (20.7)	26 (10)	2 (3.8)	0.002^b^
Affected	92 (79.3)	234 (90)	50 (96.2)	
Absence from class in this half year due to dysmenorrhea (day)				
No	113 (97.4)	215 (82.7)	32 (61.5)	< 0.001^b^
Yes	3 (2.6)	45 (17.3)	20 (38.5)	
Received menstrual education				
No	38 (32.8)	119 (45.8)	28 (53.8)	0.016^b^
Yes	78 (67.2)	141 (54.2)	24 (46.2)	
Consulted others on dysmenorrhea				
No	64 (55.2)	101 (38.8)	9 (17.3)	< 0.001^b^
Yes	52 (44.8)	159 (61.2)	43 (82.7)	
Medical consultation for dysmenorrhea				
No	110 (94.8)	241 (92.7)	48 (92.3)	0.72^b^
Yes	6 (5.2)	19 (7.3)	4 (7.7)	
Self-medication for dysmenorrhea in the last 3 months				
No	109 (94)	210 (80.8)	32 (61.5)	< 0.001^b^
Yes	7 (6)	50 (19.2)	20 (38.5)	

Data is presented as frequencies (percentages)

SD, standard deviation

^a^ one-way analysis of variance

^b^ Kruskal-Wallis test

### Health-related quality of life

No difference was reported between the average SF-36 scores of girls with and without dysmenorrhoea, except for that in role-physical (*p* = 0.04), bodily pain (*p* < 0.001), general health (*p* = 0.02), and social functioning (*p* = 0.003) domains. The comparative difference in the mental component summary between the two groups was non-significant (*p* = 0.17), whereas the difference in the physical component summary was significant (p < 0.001) after adjusting for age (Table 4). The scores in the bodily pain domain of girls with severe dysmenorrhoea were significantly lower than those of girls with mild or moderate dysmenorrhoea (Table 5).

**Table 4:** Average SF-36 scores of girls with or without dysmenorrhea

	Total Sample n = 653	With dysmenorrhea n = 428	Without dysmenorrhea n = 225	p-value (unadjusted)^a^	*p*-value (adjusted)^b^
Health-related quality of life (SF-36)					
Physical functioning	85.75 (17.97)	86.27 (16.93)	84.76 (19.82)	0.33	0.41
Role-physical	65.62 (37.70)	63.14 (38.16)	70.33 (36.45)	< 0.001	0.04
Bodily pain	64.79 (21.94)	59.58 (20.38)	74.71 (21.43)	< 0.001	< 0.001
General health	56.69 (18.33)	55.31 (18.32)	59.33 (18.10)	< 0.001	0.02
Vitality	56.37 (18.81)	55.88 (18.48)	57.31 (19.42)	0.36	0.63
Social functioning	72.48 (19.19)	70.82 (18.74)	75.64 (19.67)	< 0.001	0.003
Role-emotional	52.99 (41.90)	50.78 (41.47)	57.19 (42.48)	0.07	0.15
Mental health	60.55 (17.50)	59.77 (17.63)	62.04 (17.19)	0.11	0.15
Physical component summary (PCS) score	50.29 (7.96)	49.48 (8.03)	51.83 (7.62)	< 0.001	< 0.001
Mental component summary (MCS) score	38.39 (12.01)	37.82 (12.04)	39.54 (11.90)	0.08	0.17

Data is presented as frequencies (percentages)

^a^ Unadjusted comparisons between girls with or without dysmenorrhea were made using independent t-tests

^b^ Adjusted comparisons between girls with or without dysmenorrhea were made using linear regression with adjustment for age

**Table 5:** Severity of dysmenorrhea and mean scores of SF-36 domains

	Severity of dysmenorrheal			
	Mild dysmenorrhea (n = 116)	Moderate dysmenorrhea (n = 260)	Severe dysmenorrhea (n = 52)	p-value^a^
Health-related quality of life (SF-36)				
Physical functioning	85.56 (18.31)	86.13 (16.75)	88.56 (14.53)	0.77
Role-physical	62.07 (39.77)	63.65 (37.62)	62.98 (37.86)	0.91
Bodily pain	63.28 (19.97)	59.53 (20.40)	51.60 (21.38)	0.005
General health	57.06 (18.00)	54.76 (17.90)	54.13 (20.97)	0.44
Vitality	56.29 (16.19)	55.98 (19.10)	54.42 (20.36)	0.92
Social functioning	73.60 (17.35)	70.10 (19.40)	68.30 (17.93)	0.17
Role-emotional	47.41 (39.35)	51.92 (42.56)	52.56 (40.88)	0.60
Mental health	61.00 (15.38)	59.57 (20.40)	58.00 (21.22)	0.83
Physical component summary (PCS) score	50.05 (8.32)	49.39 (8.08)	48.71 (7.15)	0.52
Mental component summary (MCS) score	37.78 (10.61)	37.88 (12.48)	37.32 (13.00)	0.93

Data is presented as frequencies (percentages)

^a^ independent samples t-test

## Discussion

This is the first large-scale study to examine HRQOL among Chinese adolescent girls with dysmenorrhoea in Hong Kong. Results found that the prevalence of dysmenorrhoea was 65.5%, and the mean dysmenorrhoeal pain score of the girls, as measured by PVAS, was 5.43 (SD = 2.13). The majority of participants reported that dysmenorrhoea limited their daily activities. Besides, adolescent girls with dysmenorrhoea not only had a reduced quality of life in the role-physical, bodily pain and general health domains, but also in the social functioning domain compared with those without dysmenorrhoea. However, most of the girls (93.2%) did not seek medical advice despite the significant effect of dysmenorrhoea on their lives. Only approximately 18% of the participants in this study self-medicated.

### Prevalence, symptoms, and effect

The prevalence of dysmenorrhoea found in this study (65.5%) corresponded with the results of a previous local study [10]. The worldwide prevalence of dysmenorrhoea ranges from 20 to 90% [6, 8]. Different operational definitions of dysmenorrhoea may account for the differences in reported prevalence. Some empirical studies defined dysmenorrhoea as any subjective report of pain [4, 31] or pain during the previous menstrual cycle with a score higher than 5 on the 0-to-10 point PVAS [32]. The present study defined dysmenorrhoea as any subjective report of menstrual pain within the last three months. This definition is consistent with that provided by the World Health Organization [1]. The duration used to define or investigate dysmenorrhoea may also affect the reported prevalence. Ohde and colleagues revealed that only 15.8% of 823 Japanese women aged 18 to 51 years old experienced dysmenorrhoea within the previous month [33]. However, this figure was lower than that reported by another study, which reported that more than 70% of 3941 Japanese women suffered from dysmenorrhoea over the previous six months [22]. The high prevalence in that study may have been caused by recall bias given its relatively long investigation period. On the other hand, the use of a short period for reporting pain may also introduce bias because of the spontaneous resolution of symptoms [34].

The findings of this study revealed that girls with dysmenorrhoea were significantly older (*p* < 0.001) because the condition occurs when the ovulatory cycle matures [21]. The mean age at menarche of the participants in this study was 12.07 (SD = 2.23), which is close to the mean age of 12.15 reported in Singapore [35], 12.6 in Taiwan [23] and 12.0 in the United States [6] but lower than the mean age of 13.38 recently reported in Turkey [16]. These differences may be attributed to socio-economic or nutritional status and living conditions in different countries [36]. The better living conditions and nutritional status of girls in developed countries favour the early onset of menarche [36].

The mean dysmenorrhoeal pain score of the girls reported in this study echoed with previous local [10, 37] and international studies [8, 25]. Most of the girls (84%) reported that their pain localised in the lower abdomen. Participants also revealed that they experienced numerous symptoms, such as fatigue, breast tenderness and skin problems. Fatigue, headache and backache are the most common dysmenorrhoea-associated symptoms reported by 664 secondary school students in Egypt [38], whereas girls in another study reported sweating, loss of appetite and headache [39].

Over 80% of participants reported that dysmenorrhoea limited their daily activities. The results consistent with a study of 417 Taiwanese students which showed that dysmenorrhoea affects the participants’ mood (74.8%), daily activities (73.1%), academic performance (64.6%) and social life (50.1%) [11]. However, only 16% of the participants in current study missed classes within the previous half year. This result was comparatively lower than the sick leave rate of 44% reported in previous studies [6, 15]. O’Connell and colleagues reported that 39% of female students aged 19 or younger are usually absent from school for one day each month, and an additional 14% are usually absent for two or more days because of dysmenorrhoea [15]. The low sick leave rate among adolescent girls in Hong Kong may be attributed to the importance of academic achievement in Chinese society. In Chinese culture, academic achievement represents an intrinsic advancement. This attribute is particularly notable in Hong Kong, where the tight teaching schedule and stressful academic environment probably make girls reluctant to apply for sick leave [40].

### Health-related quality of life

Present findings reflected that girls perceived that not only their physical aspects but also the psychological and social aspects were affected by dysmenorrhoea. Previous studies have shown that girls commonly complain that dysmenorrhoea affects their physical aspect [16, 41]. This result is understandable because dysmenorrhoea symptoms restrict physical function. Unsal and colleagues compared the health-related quality of life (HRQOL) in Turkish university students with or without dysmenorrhoea, and the two groups exhibited differences in physical functioning, role-physical, bodily pain, general health and vitality domains. A study conducted in Australia also showed that girls with dysmenorrhoea have a lower physical functioning score in quality of life than those with other menstrual problems [17]. The results of this study likely because dysmenorrhoea primarily affects the physical health but also the daily lives and social activities of sufferers [11], thereby affecting their emotional and social functioning domains as well.

Interestingly, our findings noted that scores on the physical functioning and role-emotional domains increased with the severity of dysmenorrhoea. These differences were non-significant, indicating that girls with severe dysmenorrhoea were likely to have a better quality of life in these aspects. One possible explanation is that girls with severe dysmenorrhoea receive more concern from other people or from their friends or mothers. In particular, in Chinese culture, mothers are responsible for their daughters and manage all aspects of their care [42]. As a result, daughters who suffer from dysmenorrhoea would receive constant concern from their mothers, thus receiving better emotional support.

### Medical consultation and self-medication

Most of the girls (93.2%) did not seek medical advice despite the significant effect of dysmenorrhoea on their lives. Local and international studies reported similarly low rates for medical consultation among females with dysmenorrhoea [10, 35, 37, 38]. The impression that many girls experience pain may reinforce the concept of normality, which in turn discourages girls from questioning the significance of their pain and considering medical consultation [43]. Only approximately half the participants in this study had received menstrual education, reflecting the inadequacy of the education provided to them and the likelihood that insufficient knowledge on the physiology of menstruation contributes to the low rate of medical consultation [10]. Previous studies in Australia and England have shown that women fail to seek medical help for menstrual symptoms because they encounter difficulty in differentiating between normal and pathological symptoms [44, 45]. All these factors suggest the importance of not only educating girls about what constitutes an “abnormal” menstrual period, but also of the need for open conversation about dysmenorrhoea to raise public awareness of its problems.

Only approximately 18% of the participants in this study self-medicated. By contrast, previous studies have reported moderate to high rates of self-medication among adolescent girls with dysmenorrhoea (60 to 80%) [19, 32]. The result of the present study is in line with a study that reported that local secondary school girls seldom take analgesics to manage dysmenorrhoea [10]. In addition, the present findings further support previous studies maintaining that self-medication is not part of the construct of self-care among adolescent Chinese girls with dysmenorrhoea [46–48]. The low rate of self-medication may be attributed to the perception of girls that self-medication require a prescription or at least medical advice and should not be self-initiated. Without a prescription, girls would not take medication or buy it from a pharmacy. This interpretation explains why self-medication is not relevant to the self-care construct among adolescent girls with dysmenorrhoea.

Although relatively few girls self-medicated for dysmenorrhoea, those who did used medication inappropriately. Some girls reported taking three or ‘a few’ paracetamol tablets each time they experienced pain, and one girl mentioned using famotidine, a drug that inhibits stomach acid production, to relieve dysmenorrhoea. Others could not name what medication they took. Girls did not know the medication they used, used it inconsistently or took less than the recommended dose. These behaviours imply that girls lacked knowledge of self-medication and raise the possibility of adverse health effects of inappropriate self-medication [15, 35]. The findings highlighted the need for improving the understanding of self-medication among adolescent girls in Hong Kong. They also shed light on the need to develop and provide appropriate educational interventions related to dysmenorrhoea, with the correct administration of self-medication as an essential component. Girls should also be reminded to use self-medication only after receiving proper education.

### Strengths and limitations

The strengths of this study included examining the quality of life of Chinese adolescent girls with dysmenorrhoea, whereas previous studies mainly focused on Western university students or other women [16, 19] or else only examined the influence of dysmenorrhoea on the daily lives and school performance of adolescents but not their quality of life [19, 27]. This study also expands previous findings that compare the quality of life of Chinese girls with or without dysmenorrhoea and with different severity levels of the condition. However, the results of the current study should be interpreted with caution given its limitations. First, girls were recruited from secondary schools because a school-based sample is convenient for a researcher who wants to recruit a large number of participants within a short period of time. However, such a sample could not represent girls who left or dropped out of school. This recruitment method can only reasonably be expected to represent the majority of, but not all, adolescent girls in Hong Kong. Second, this study excluded secondary dysmenorrhoea on the basis of the participants’ responses. However, dysmenorrhoea was not clinically verified. Although only nearly 10% of girls have severe secondary dysmenorrhoea, the current study did not employ ultrasound or pelvic examination to prove the absence of secondary dysmenorrhoea during the recruitment process given their expense [49]. The possibility of including a girl with a secondary cause of dysmenorrhoea cannot be completely avoided, but girls who reported a history of gynecological disease (such as endometriosis, ovarian cysts or pelvic inflammation) or gynecological surgery, which are both potential causes of secondary dysmenorrhoea [50], were excluded from this study. Nevertheless, as a history of fever during periods is a good way to exclude pelvic inflammatory disease or pathology and thus can be used to differentiate between primary and secondary dysmenorrhoea, future studies should ask girls if they experienced fevers during periods. Finally, data were collected from retrospective self-reports, perhaps leading to recall bias. In addition, the retrospective nature of self-reporting may amplify the cyclicity of pain variation. Nevertheless, retrospective reports of pain severity have a reasonable standard of accuracy [51].

### Implications to clinical practice

The findings of this study expand understanding of HRQOL of adolescent Chinese girls with dysmenorrhoea. Healthcare professionals should be made aware that adolescent girls with dysmenorrhoea are likely to have a lower quality of life. Screening should be carried out for the early identification of girls with dysmenorrhoea and for timely interventions. In addition, a brief educational intervention to enhance knowledge of appropriate medications could reduce the severity of dysmenorrhoea and enhance the quality of life among adolescent girls [52]. Clearly, effective educational interventions should be developed and provided to this group of girls.

## Conclusions

This study confirms the findings of previous works showing that dysmenorrhoea is a widespread problem and identifies its detrimental effect on adolescent girls. The consequences of dysmenorrhoea appear to be substantial. Thus, adolescent girls should receive assistance to manage and reduce the effects of dysmenorrhoea. Effective interventions for symptom relief should be identified and provided to this vulnerable group to improve their quality of life. Such interventions would not only benefit adolescent girls but society as a whole.

## Abbreviations

HRQOL: health-related quality of life
PVAS: The Pain Visual Analogue Scale
SF-36: Short-Form Health Survey
